# Small Lymphocytic Lymphoma/Chronic Lymphocytic Leukemia Presenting as a Nasopharyngeal Mass With Bilateral Otitis Media With Effusion: A Case Report

**DOI:** 10.7759/cureus.95459

**Published:** 2025-10-26

**Authors:** Ghezlan Aldawas, Imtiyaz N Bhat, Sulaiman Alhamad

**Affiliations:** 1 Otolaryngology - Head and Neck Surgery, Al-Farwaniya Hospital, Kuwait City, KWT; 2 Otolaryngology - Head and Neck Surgery, Ministry of Health, Kuwait, Kuwait City, KWT; 3 Otolaryngology - Head and Neck Surgery, Al-Farwaniya Hospital, Kuwait, KWT

**Keywords:** case report, chronic lymphocytic leukemia, nasopharynx, otitis media with effusion, small lymphocytic lymphoma

## Abstract

Small lymphocytic lymphoma/chronic lymphocytic leukemia (SLL/CLL) is an indolent B-cell malignancy that rarely involves the nasopharynx. Such cases may present with nonspecific ear, nose, and throat (ENT) symptoms, delaying diagnosis.

In this case report, we present a 54-year-old male who presented with a six-month history of nasal obstruction and bilateral hearing loss due to otitis media with effusion. Nasoendoscopy demonstrated a nasopharyngeal mass. He underwent biopsy and excision of the nasopharyngeal mass, bilateral myringotomy with grommet tube insertion, and turbinoplasty. Histology and immunohistochemistry (IHC) confirmed SLL/CLL. Positron emission tomography (PET)/ computed tomography (CT) showed cervical, axillary, and inguinal lymphadenopathy. Molecular studies found an immunoglobulin heavy chain variable mutation (IGHV) with wild-type TP53. The patient was started on Zanubrutinib 160 mg twice daily, resulting in a rapid resolution of symptoms.

SLL and CLL presenting as a nasopharyngeal mass are very rare and can be challenging for ENT experts. Adults with persistent otitis media with effusion (OME) and a nasopharyngeal mass should undergo biopsy with IHC and molecular testing to avoid underdiagnosis and ensure timely targeted therapy.

## Introduction

Small lymphocytic lymphoma (SLL) and chronic lymphocytic leukemia (CLL) are mature B-cell neoplasms. They represent a single underlying pathophysiology, but two distinct clinical entities. The fifth edition of the World Health Organization’s (WHO) classification and the International Consensus Classification (ICC) considers a diagnosis of CLL if the patients present with leukemia and a diagnosis of SLL in case the patients present with masses in lymph nodes or otherwise, not with the presence of significant lymphocytosis [1,2]. The two conditions, however, have different clinical courses [3,4]. In addition to the nodes, the conditions may involve other organs like the skin, kidneys, and lungs [1,4].

In contrast, nasopharyngeal lymphomas exhibit a unique histological distribution. The commonest subtypes include diffuse large B-cell lymphoma (DLBCL) and the nasal type of extranodal NK/T-cell lymphoma (ENKTCL) [5]. The latter, having a strong association with Epstein-Barr virus (EBV), occurs mostly in the nasal cavity and structures present in the midline of the face, while the former is the most common B-cell nasopharyngeal pathology [6]. The 2016 WHO classification and its 2022 update have further refined this disease concept [7]. Such data highlight the unusual nature of a diagnosis of SLL/CLL in the nasopharyngeal region.

This rarity has practical implications for otolaryngologists, as nasopharyngeal involvement by hematologic malignancies can mimic common benign ENT conditions. Nevertheless, research has shown that malignant conditions of the nasopharynx can manifest with nonspecific ear, nose, and throat symptoms like nasal obstruction, neck swelling, hearing loss, and ear fullness [5,6]. The International Workshop on CLL (iwCLL) guidelines highlight the importance of standardized diagnostic criteria and indications for treatment [8]. 

In this case report, we present a 54-year-old male with a nasopharyngeal mass and bilateral otitis media with effusion, initially managed by the otolaryngology team. This case highlights the diagnostic challenge of SLL/CLL presenting in the nasopharynx and underscores the importance of biopsy, immunohistochemistry, and molecular characterization within the WHO/ICC framework for accurate diagnosis and targeted management. 

## Case presentation

A 54-year-old male, a retired police officer with a medical history of hypertension and hyperlipidemia, presented with a six-month history of progressive nasal obstruction associated with gradually worsening bilateral hearing loss and intermittent aural fullness. He denied fever, night sweats, weight loss, epistaxis, or dysphagia, and had no history of trauma, previous nasal surgery, or malignancy. His family and social history were unremarkable.

On examination, anterior rhinoscopy revealed hypertrophy of the inferior turbinates, while flexible nasopharyngoscopy demonstrated a smooth, fleshy mass occupying the nasopharyngeal vault. Otoscopic examination showed bilateral middle ear effusion, and pure tone audiometry confirmed bilateral conductive hearing loss with thresholds of 55 dB in the left ear and 50 dB in the right.

On 14 April 2025, the patient underwent excision of the nasopharyngeal mass, bilateral myringotomy and grommet tube insertion, along with bilateral turbinoplasty. Intraoperatively, the mass appeared soft, fleshy, and non-ulcerated. Hemostasis was achieved, and the excised specimens were sent for histopathological analysis.

**Figure 1:**
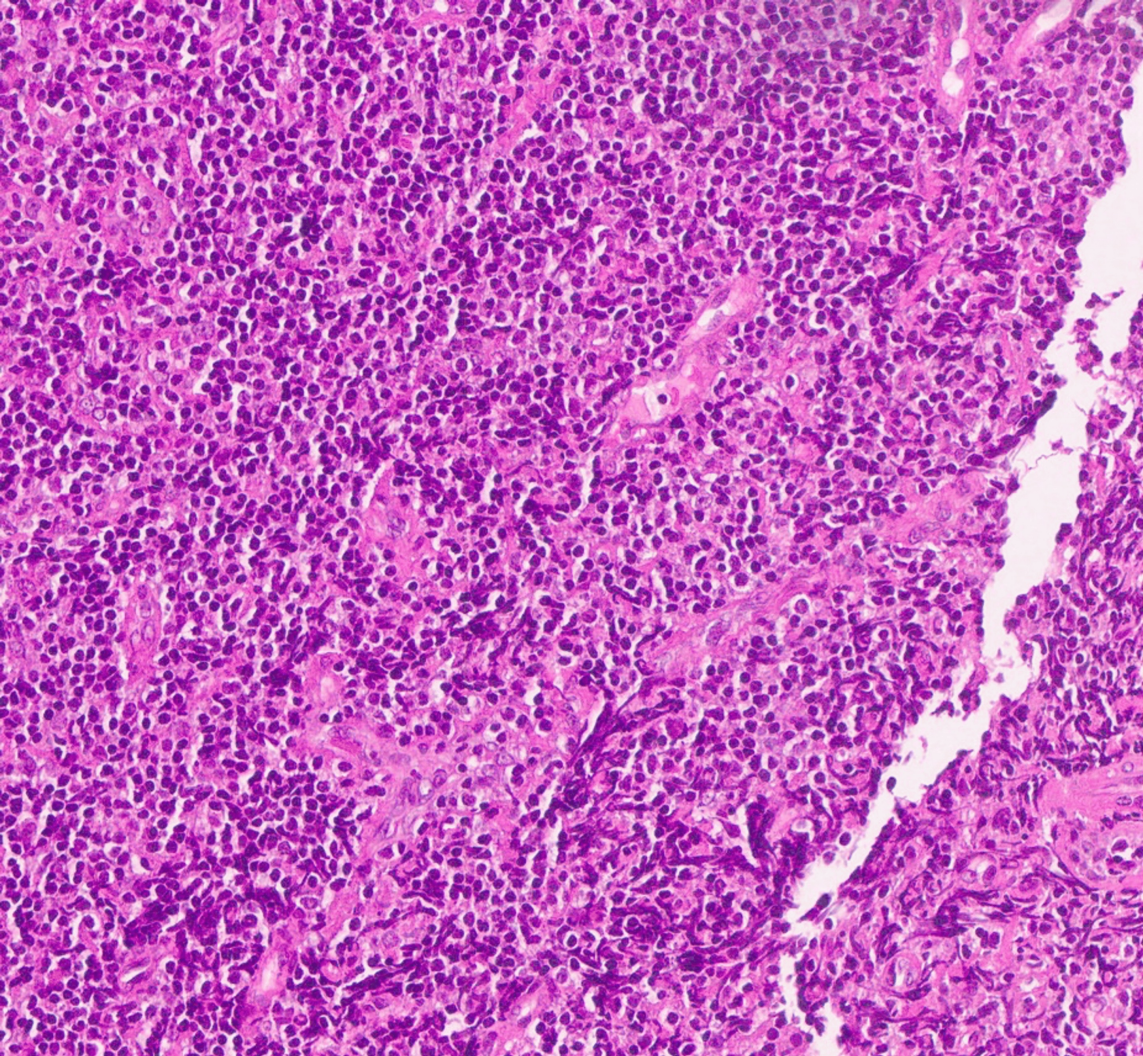
Hematoxylin and Eosin stain showing effaced lymphoid architecture by small mature lymphocytes (×400)

**Figure 2:**
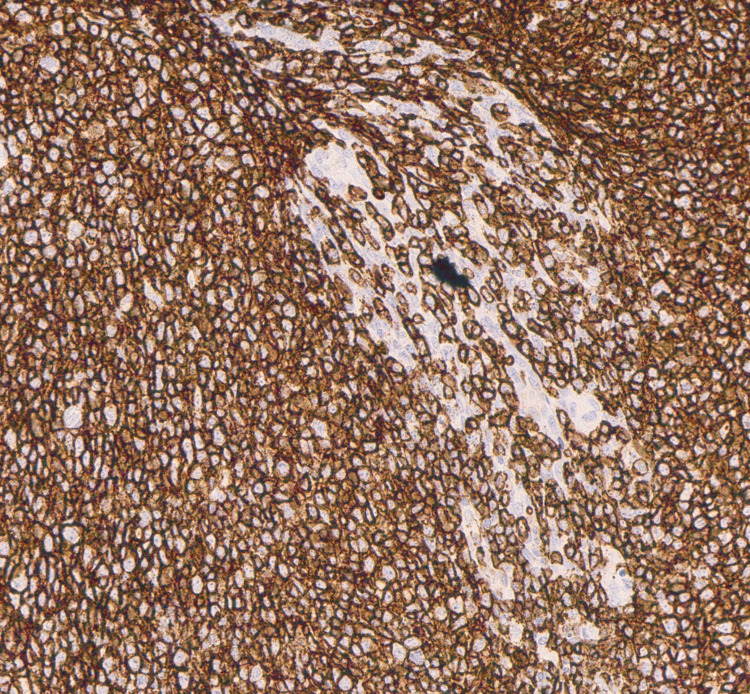
Immunohistochemistry (IHC) showing diffuse CD20 positivity (×400)

**Figure 3:**
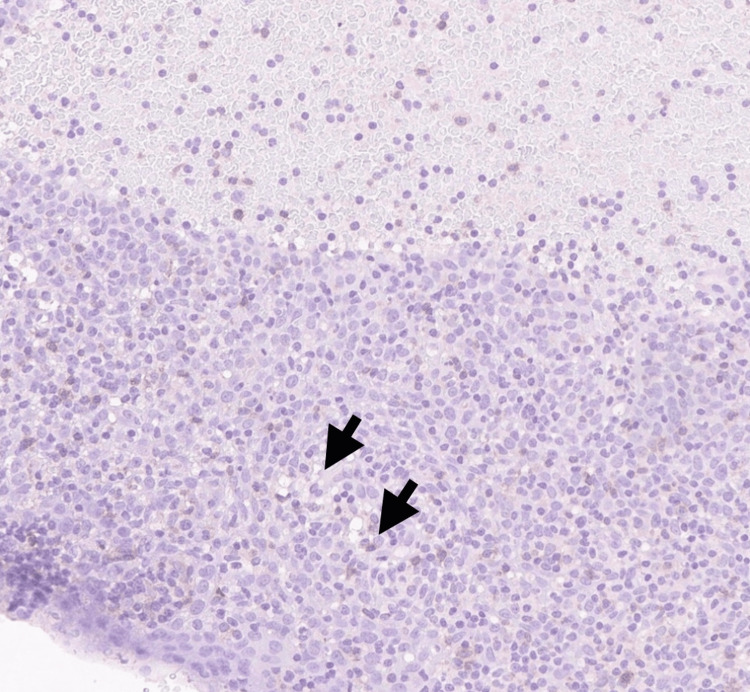
Immunohistochemistry (IHC) showing CD5 co-expression in tumor cells (x400) Arrows highlight tumor cells with membranous CD5 positivity

**Figure 4:**
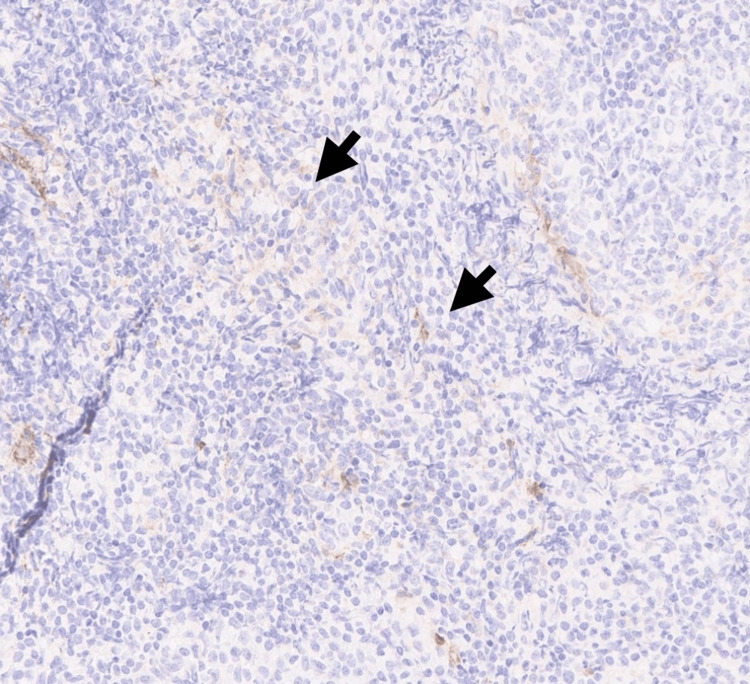
Immunohistochemistry (IHC) demonstrating variable CD23 expression (x400) Arrows indicate scattered tumor cells showing weak to moderate membranous CD23 positivity

**Figure 5:**
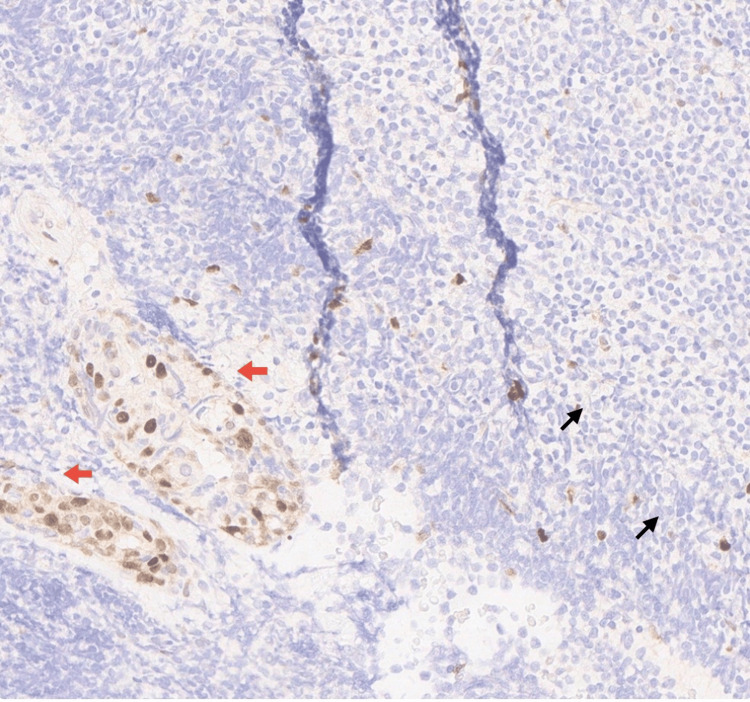
Immunohistochemistry (IHC) negative for Cyclin D1, excluding mantle cell lymphoma (x400) ( Black arrows ) indicate Cyclin D1- negative tumor cells, while ( Red arrows ) highlight adjacent glandular structures showing positive internal control staining.

Histopathology revealed effacement of the lymphoid architecture by small mature lymphocytes with clumped chromatin and scattered proliferation centers, as shown in Figure 1. Immunohistochemistry demonstrated diffuse positivity for CD20 and CD5 (Figures 2, 3) and Bcl-2, with variable CD23 expression (Figure 4), and was negative for CD3, CD10, and Cyclin D1 (Figure 5). These morphological and immunophenotypic findings (Figures 1-5) were consistent with small lymphocytic lymphoma/chronic lymphocytic leukemia (SLL/CLL).

Hematology evaluation demonstrated 9% clonal B-CLL cells in the peripheral blood. Molecular studies confirmed immunoglobulin heavy chain variable mutation (IGHV) with wild-type TP53. Fluorescence in situ hybridization (FISH) was negative for del(17p) and t(11;14). Blood counts showed hemoglobin of 9.5 g/dL (reference range: 13-17 g/dL), white cell count of 12,000/µL (4,000-11,000/µL), lymphocytes 2,000/µL (1,000-4,800/µL), neutrophils 6,000/µL (1,500-8,000/µL), and platelets 177,000/µL (150,000-400,000/µL). Lactate dehydrogenase (LDH), erythrocyte sedimentation rate (ESR), and albumin levels were within normal limits.

**Figure 6:**
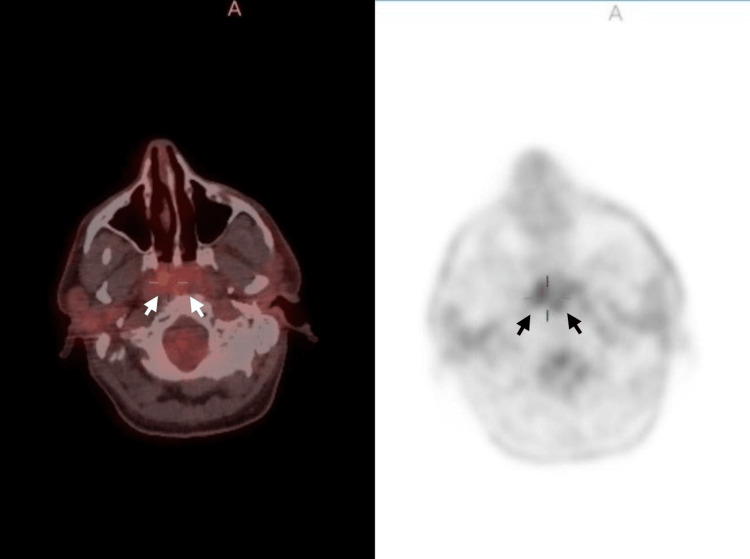
PET and fused PET/CT axial images demonstrating FDG uptake at the nasopharyngeal surgical bed, with higher uptake on the right side (SUVmax 4.2) and milder uptake on the left side (SUVmax 3.4)

A PET/CT scan performed on 2 May 2025 demonstrated mild fluorodeoxyglucose (FDG) uptake at the surgical bed with standardized uptake value (SUV)max 4.2 on the right and 3.4 on the left, as well as multiple mildly hypermetabolic cervical, axillary, and inguinal lymph nodes. No extranodal organ involvement was identified. The radiologist considered the findings most consistent with postoperative inflammatory change, although residual disease could not be excluded (Figure 6).

Following multidisciplinary team discussion, the patient was commenced on Zanubrutinib 160 mg twice daily on 27 June 2025. Prophylaxis with acyclovir 400 mg twice daily and co-trimoxazole 480 mg twice daily on Mondays, Wednesdays, and Fridays was initiated, along with a first cycle of allopurinol. The patient tolerated treatment well, with no atrial fibrillation or major adverse events. Mild alanine aminotransferase (ALT) elevation was noted (65 U/L; normal range 7-56 U/L), while electrocardiogram (ECG) confirmed a normal corrected QT interval (QTc).

Clinically, there was rapid improvement, with resolution of nasal obstruction and hearing loss. Follow-up endoscopy and audiology confirmed clearance of middle ear effusions. The patient remains on Bruton tyrosine kinase (BTK) inhibitor therapy with hematology follow-up every one to two months and repeat imaging planned at six months (January 2026).

## Discussion

SLL and CLL are indolent mature B-cell lymphoproliferative neoplasms that represent a rare subset of small B-cell malignancies. Within the sinus and nasopharynx, the lymphomas observed by ENT surgeons are mainly DLBCL, while ENKTCL and indolent small B-cell conditions such as SLL/CLL are far less frequent and usually limited to sporadic cases [6,9,10]. Clinically, these rare conditions create a diagnostic pitfall for ENT specialists. This case highlights that in adults, particularly when persistent otitis media with effusion (OME) is accompanied by a nasopharyngeal mass, nasopharyngoscopy and biopsy must be performed promptly to avoid delays in diagnosis [11,12].

The differential diagnosis of nasopharyngeal lymphomas is dominated by aggressive histologies such as DLBCL and NK/T-cell lymphoma. A diagnosis of SLL/CLL is established through correlation of morphology and immunophenotype [6,10], typically showing CD5 and CD23 co-expression with negativity for Cyclin D1 and CD10, while lymphoid enhancer-binding factor 1 (LEF1) positivity further supports the diagnosis [9,10,13].

From a prognostic perspective, mutated IGHV and the absence of TP53 disruption are associated with more favorable outcomes, whereas del(17p) or TP53 mutation predicts refractoriness to chemoimmunotherapy and inferior prognosis [9,14]. Current recommendations emphasize formal TP53 testing, both by FISH for del(17p) and sequencing for mutations, at diagnosis and before each new line of therapy, since results directly inform treatment selection [14]. These biologic principles apply equally when the disease is first recognized by ENT specialists, as accurate pathology and molecular profiling guide downstream hematology management [9].

In this case, the favorable molecular profile (mutated IGHV and wild-type TP53) supported the use of Zanubrutinib as first-line therapy, consistent with current iwCLL and European Society for Medical Oncology (ESMO) recommendations [4,12]. The patient’s rapid clinical response further supports the efficacy of targeted therapy in indolent B-cell lymphomas presenting in the nasopharynx. 

Few similar cases have been reported in the literature, emphasizing the rarity of nasopharyngeal SLL/CLL and reinforcing the importance of early biopsy, molecular testing, and ENT-hematology collaboration in achieving accurate diagnosis and timely treatment.

This case provides several learning points. Adult patients with persistent OME and a nasopharyngeal mass should undergo biopsy, as labeling such symptoms as benign without tissue testing risks misdiagnosis [11,12]. A comprehensive diagnostic panel including CD5, CD23, cyclin D1, CD10, and optionally LEF1, along with molecular work-up for del(17p), TP53 sequencing, and IGHV status, is necessary to avoid diagnostic errors [6,9,10,13,14]. Prompt referral to hematology is crucial, as targeted agents such as Zanubrutinib offer excellent therapeutic efficacy and rapid symptom control.

Our observation is limited to a single patient with relatively short follow-up at submission, which constrains generalizability. Nevertheless, this case adds to the scarce literature on nasopharyngeal SLL/CLL and highlights practical ENT steps: early biopsy, full immunohistochemical and molecular profiling, and timely hematology co-management that can expedite definitive care [6,10]. From the patient perspective, individuals commonly describe frustration with “recurrent ear blockage” before diagnosis; clear communication about the need for biopsy and the possibility of malignant pathology can improve the diagnostic journey [11,12].

## Conclusions

In conclusion, Small lymphocytic lymphoma (SLL) and chronic lymphocytic leukemia (CLL) are indolent B-cell neoplasms that rarely involve the nasopharynx and can be challenging for ENT experts. All adults with persistent OME and nasopharyngeal mass should undergo a biopsy, followed by immunohistochemistry and molecular testing, to avoid underdiagnosis.

This case emphasizes the importance of considering lymphoma in the differential diagnosis of persistent adult OME with a nasopharyngeal mass and highlights the role of a multidisciplinary approach involving ENT, pathology, radiology, and hematology in optimizing patient outcomes. Early recognition and coordinated management are crucial to prevent delays in care and improve prognosis. Continued research and clinical collaboration are essential to enhance our understanding and management of this complex condition.
